# Quantitative Proteomics Analysis of Herbaceous Peony in Response to Paclobutrazol Inhibition of Lateral Branching

**DOI:** 10.3390/ijms161024332

**Published:** 2015-10-14

**Authors:** Daqiu Zhao, Saijie Gong, Zhaojun Hao, Jiasong Meng, Jun Tao

**Affiliations:** Jiangsu Key Laboratory of Crop Genetics and Physiology, College of Horticulture and Plant Protection, Yangzhou University, Yangzhou 225009, China; E-Mails: dqzhao@yzu.edu.cn (D.Z.); gong.sai.jie@163.com (S.G.); haozhaojunko@126.com (Z.H.); jsmeng@yzu.edu.cn (J.M.)

**Keywords:** herbaceous peony, paclobutrazol application, proteomics analysis, iTRAQ

## Abstract

Herbaceous peony (*Paeonia lactiflora* Pall.) is an emerging high-grade cut flower worldwide, which is usually used in wedding bouquets and known as the “wedding flower”. However, abundant lateral branches appear frequently in some excellent cultivars, and a lack of a method to remove *Paeonia lactiflora* lateral branches other than inefficient artificial methods is an obstacle for improving the quality of its cut flowers. In this study, paclobutrazol (PBZ) application was found to inhibit the growth of lateral branches in *Paeonia lactiflora* for the first time, including 96.82% decreased lateral bud number per branch, 77.79% and 42.31% decreased length and diameter of lateral branches, respectively, declined cell wall materials and changed microstructures. Subsequently, isobaric tag for relative and absolute quantitation (iTRAQ) technology was used for quantitative proteomics analysis of lateral branches under PBZ application and control. The results indicated that 178 differentially expressed proteins (DEPs) successfully obtained, 98 DEPs were up-regulated and 80 DEPs were down-regulated. Thereafter, 34 candidate DEPs associated with the inhibited growth of lateral branches were screened according to their function and classification. These PBZ-stress responsive candidate DEPs were involved in eight biological processes, which played a very important role in the growth and development of lateral branches together with the response to PBZ stress. These results provide a better understanding of the molecular theoretical basis for removing *Paeonia lactiflora* lateral branches using PBZ application.

## 1. Introduction

Herbaceous peony (*Paeonia lactiflora* Pall. (*P. lactiflora*)) is an emerging high-grade cut flower worldwide, which is usually used in wedding bouquets and known as the “wedding flower” [1]. As an ideal cut flower, it not only requires excellent flower shape, flower color, flower fragrance, inflorescence stem, leaf and the performance of vase, but also possesses the property of few or no lateral branches. However, the more lateral branches have hampered the cutting flower production of Chinese *P. lactiflora* cultivars. These lateral branches not only consume plant nutrition but also affect the growth and development of the terminal buds, thereby further worsening the quality of cut flowers. Therefore, the lateral branches should be removed as soon as possible in the process of *P. lactiflora* cut flower production, which plays an important role in enhancing the cut flower quality of *P. lactiflora*, such as prolonging the full opening stage of flowers as well as increasing the flower diameters, the thickening rate, and elongation rate of inflorescence stems [2]. However, only artificial methods have been used to remove *P. lactiflora* lateral branches until now, which are inefficient, require a lot of labor and time, and have a negative impact on the large-scale production. Therefore, exploring a method to inhibit the growth of *P. lactiflora* lateral branches is of considerable significance.

Paclobutrazol (PBZ), a triazole compound, is widely used as a growth retardant for controlling vegetative growth in a wide range of horticultural plants [3,4,5,6]. In *P. lactiflora*, Wang *et al.* [7] has also studied the effect of PBZ on the growth of five cultivars, their plant heights and crown breadths were reduced, the plant stem diameters were increased, and 100 mg/L concentration PBZ had the best integrated effect. In addition to the above-mentioned effects, we also found that PBZ could significantly inhibit the growth of *P. lactiflora* lateral branches and reduce lateral buds to blossoming out, which was rarely seen in the existing literature. This finding would greatly save on the labor and time needed for removing *P. lactiflora* lateral branches, and reduce the cost and difficulty of the production and management.

Until now, it has been generally believed that the formation of plant lateral branches involves two processes: one is the initiation of the lateral buds, and the other is the growth and development of the lateral buds. After the formation of lateral bud primordiums, they begin with the elongation growth to form lateral buds, and then continue to develop into the lateral branches [8]. Meanwhile, the growth and development of lateral branches is believed to be linked to plant hormones, including auxin (IAA), cytokinin (CK) and strigolactone (SL), and a large number of molecular biology studies have been performed around them [8]. For example, in the biosynthetic pathway of strigolactone, a lot of genes including *dwarf27* (*D27*) [9], *D14* [10], *teosinte branched1* (*TB1*) [11], *branched1* (*BRC1*) [12,13] and *fine culm1* (*FC1*) [14] have been identified as associated with the growth and development of lateral branches. However, the formation and development of plants’ characters is often controlled by numerous genes; thus, it is very difficult to fully clarify the inherent mechanism of its development and regulation only from the perspective of hormones. As a powerful technique to perform quantitative proteome analysis, isobaric tag for relative and absolute quantitation (iTRAQ) can shed light on the inherent mechanism of the growth and development of lateral branches [15]. Compared with the two-dimensional-electrophoresis-based approaches, iTRAQ has many advantages including high identification rate of proteins, accurate quantification of different proteins, and high reproducibility [16,17]. This technique has been widely applied in a number of studies, such as fiber differentiation and initiation of cotton [18], and wheat grain development [15]. However, the application of iTRAQ in the growth and development of lateral branches is little known. In this study, to illustrate the molecular metabolism of PBZ inhibiting the growth of *P. lactiflora* lateral branches, proteomics analysis of the lateral branches (except leaves and buds) under control and PBZ application was performed using iTRAQ. A greater understanding of this information can provide a theoretical basis to breed ideal *P. lactiflora* cut flower varieties without lateral branches.

## 2. Results

### 2.1. Morphological Indices

**Figure 1:**
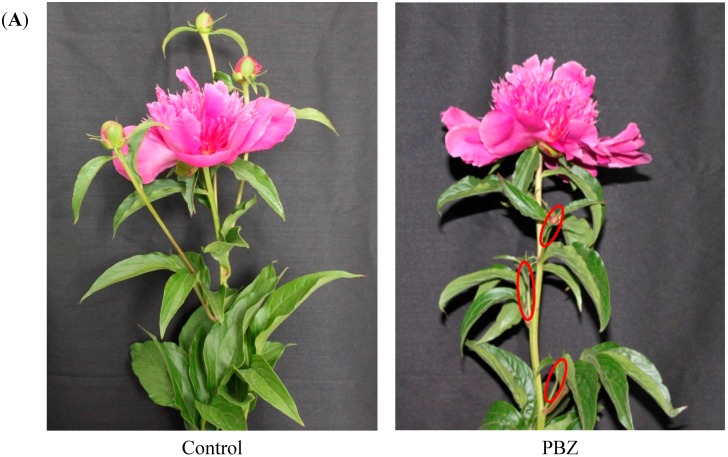

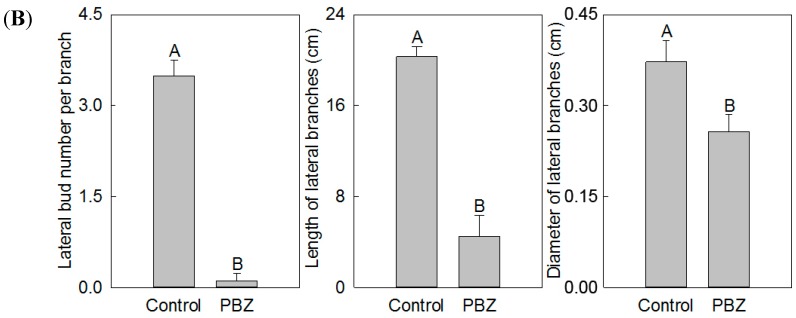
Effect of PBZ application on morphology of *P. lactiflora* lateral branches: (**A**) Photographs of *P. lactiflora* lateral branches; (**B**) statistics of morphological indices in *P. lactiflora* lateral branches. Three biological replications with each 100 plants were used to perform the statistical analysis. Red circles indicate *P. lactiflora* lateral branches under PBZ application. Different letters indicate highly significant differences (*p* < 0.01). PBZ: paclobutrazol.

The effect of PBZ on the morphology of *P. lactiflora* lateral branches was first investigated in this study (Figure 1). In the full-bloom stage, there were 3.49 lateral buds in a single branch of control, and after PBZ application, the lateral bud number per branch was significantly decreased by 96.82%, the length and diameter of lateral branches were also significantly lower than those of the control with 77.79% and 42.31%, respectively.

### 2.2. Microstructures and Cell Wall Materials

**Figure 2:**
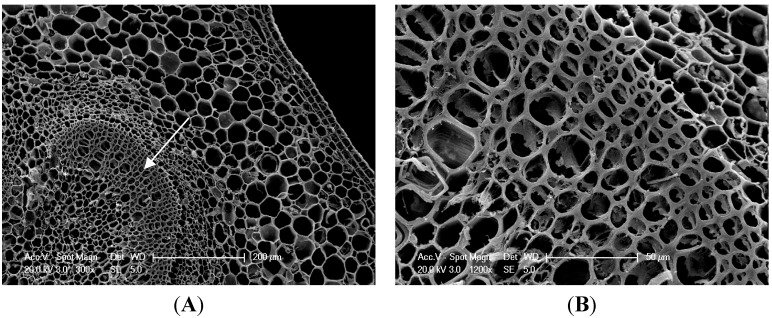

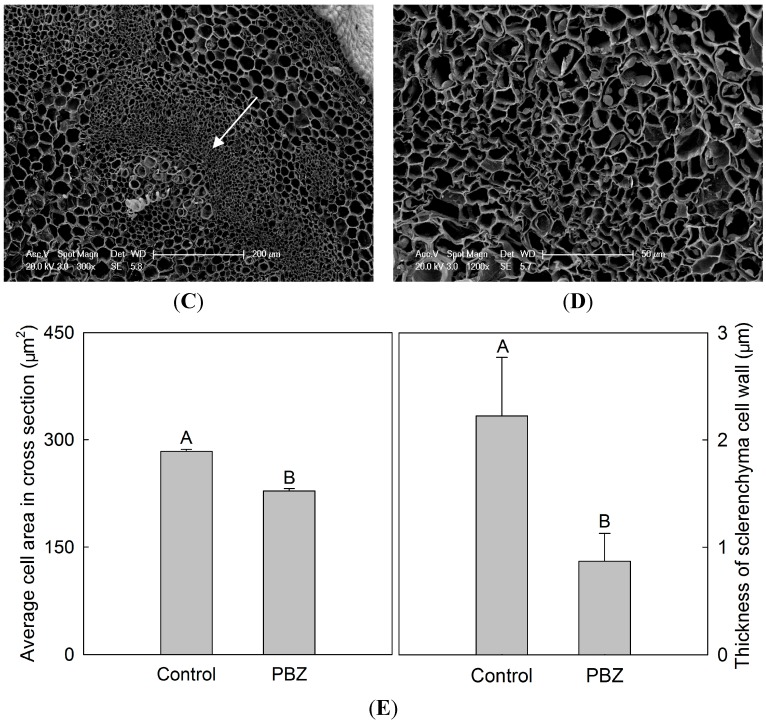
Effect of PBZ application on microstructure of *P. lactiflora* lateral branches: (**A**,**C**) Photographs of *P. lactiflora* lateral branches with a magnification of 200 times (Scale bar = 200 μm); (**B**,**D**) Photographs of partial enlargement in (**A**,**C**) marked by the arrow. Scale bar = 50 μm; and (**E**) Statistics of microstructure in *P. lactiflora* lateral branches. Different letters indicate highly significant differences (*p* < 0.01).

The microstructures of lateral branches are intuitively shown in Figure 2A–D. Compared with the control, the deformation of sclerenchyma cells was observed and its cell walls had not been thickened under PBZ application. By micrometer measurement, the average cell area and the thickness of the sclerenchyma cell walls in a cross section of the lateral branches under PBZ application were significantly decreased by 19.54% and 60.86%, respectively (Figure 2E).

**Figure 3:**
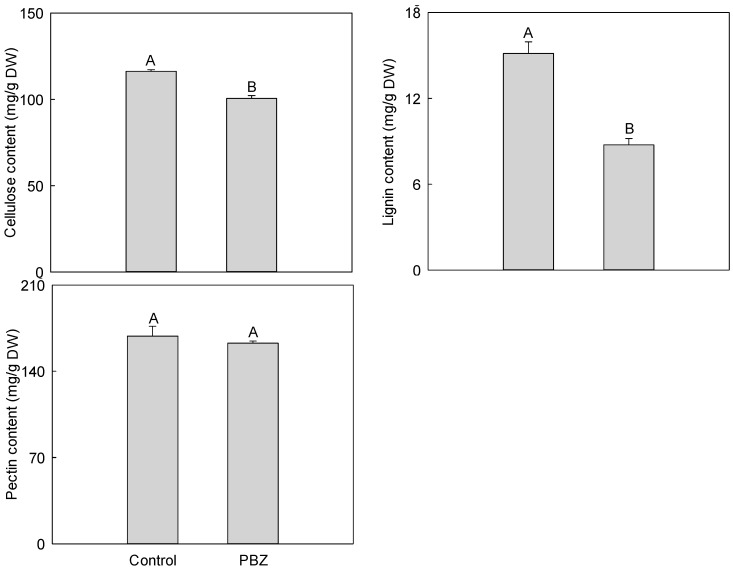
Effect of PBZ application on cell wall materials of *P. lactiflora* lateral branches. Different letters indicate highly significant differences (*p* < 0.01).

Cellulose and lignin were the major cell wall components, their contents under PBZ application were all lower than those of the control with 13.51% and 42.18%, respectively, and their differences all reached highly significant levels. In addition, the pectin content under PBZ application was also decreased by 3.36%, but the difference was not significant (Figure 3).

### 2.3. Protein Identification

**Figure 4:**
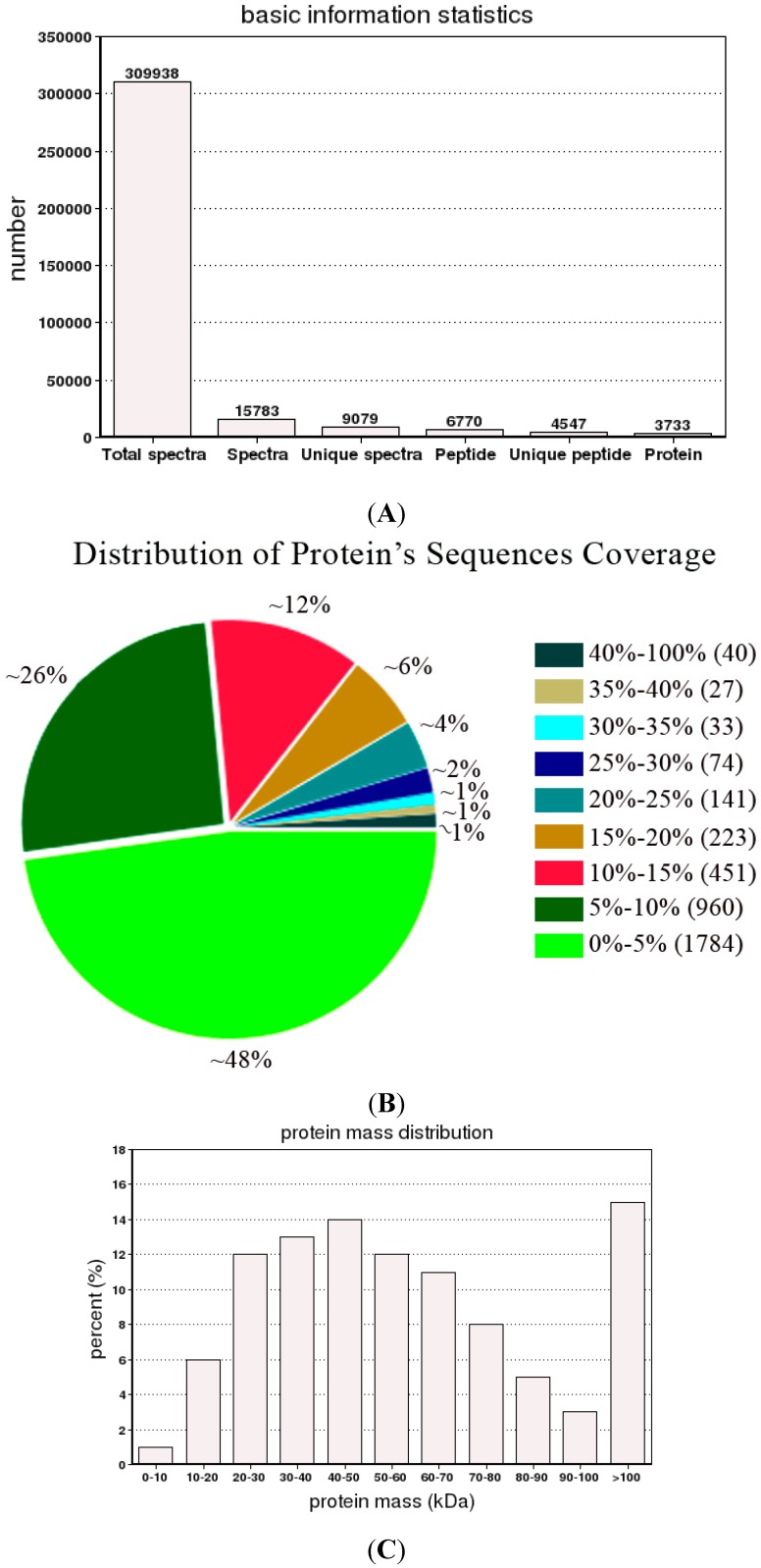

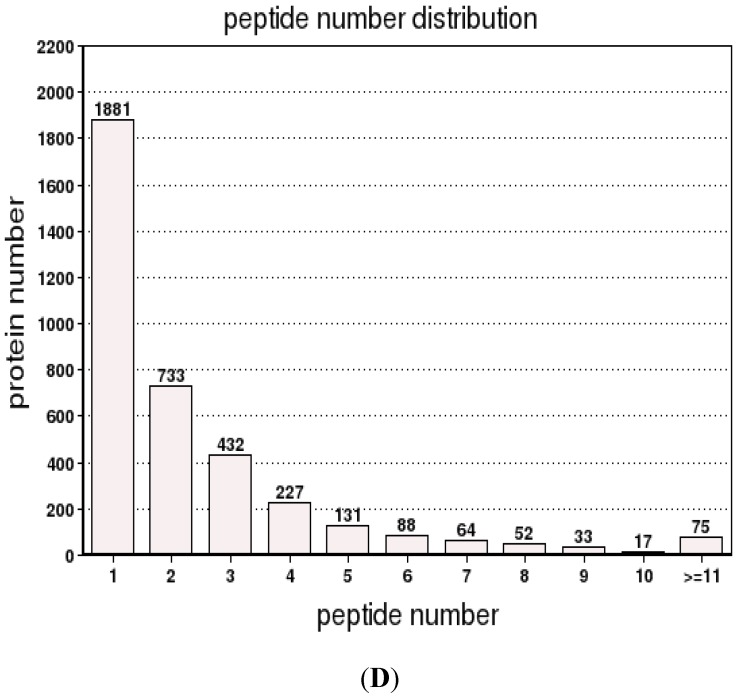
The basic information of protein mass spectrometry in *P. lactiflora* lateral branches by iTRAQ: (**A**) basic information statistics; (**B**) distribution of protein’s sequences coverage; (**C**) protein mass distribution; and (**D**) peptide number distribution. iTRAQ: isobaric tag for relative and absolute quantitation.

In order to deeply explore the mechanism of PBZ inhibiting the growth of *P. lactiflora* lateral branches, proteomics analysis was performed by iTRAQ. Firstly, the total proteins of *P. lactiflora* lateral branches under control and PBZ application were extracted. Subsequently, mass spectrometry (MS) was used to identify these proteins according to a Triple TOF5600, and the error distribution of the peptide spectra match quality was adopted to assess its identified quality. Errors over 0.05 Da were defined as false positives. The error distribution of the proteomic identification in *P. lactiflora* lateral branches is shown in Figure S1, which was mainly concentrated between −10 and 10 ppm, indicating that the identified result was relatively good. Furthermore, the basic information of the protein MS according to iTRAQ was analyzed. Overall, 4547 unique peptides and corresponding to 3733 proteins were identified (Figure 4A, Table S1). The distribution of identified protein sequences coverage showed that the number of proteins was basically presented a declining trend with increased coverage (Figure 4B). However, the protein mass distribution was not uniform, and most of the protein molecular weights were concentrated on 20–70 kDa (Figure 4C). In addition, the protein number was basically decreased with the increased number of matching peptides, and the peptide number in most of identified proteins was within 10 (Figure 4D). In addition, the functional annotation and classification of these identified proteins was showed in Figure S2 and Figure S3, Table S2 and Table S3.

### 2.4. Comparative Analysis of DEPs between Control and PBZ Application

**Figure 5:**
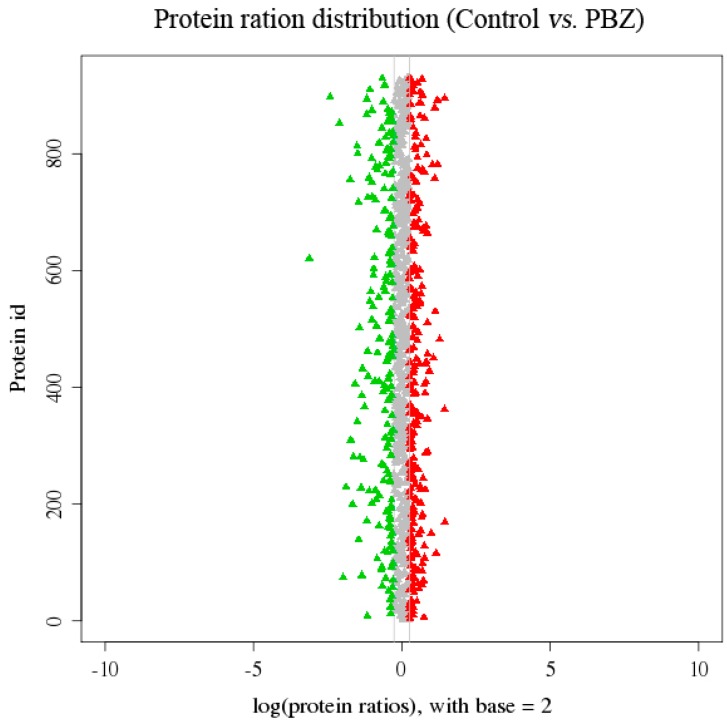
The distribution of protein abundance quantified in *P. lactiflora* lateral branches. The protein would be marked with red or green if protein ratios >1.2; red scatters indicate up-regulated DEPs and green scatters indicate down-regulated DEPs.

To identify candidate differentially expressed proteins (DEPs) controlling the growth of *P. lactiflora* lateral branches, the distribution of the protein abundance was analyzed, and a 1.2-fold cut-off was used to implicate significant changes in the abundance of DEPs (Figure 5, Table S4 and Table S5). Of the 3733 identified proteins, 178 showed more than 1.2-fold changes with *p*-value < 0.05 in the protein expression level. Among these 178 proteins, 98 DEPs were up-regulated (red scatters) and 80 DEPs were down-regulated (green scatters). Furthermore, the pathway analysis in Kyoto Encyclopedia of Genes and Genomes database (KEGG) was illustrated to help us understand this result, and 165 DEPs (92.70%) were assigned to 74 KEGG pathways (Table S6). According to the *p*-value ≤ 0.05 and DEPs ≥ 5, only nine KEGG pathways were obtained involving large amounts of substance metabolism processes. Among them, metabolic pathways contained the largest number of DEPs (90 DEPs, 54.55%, ko01100), with the biosynthesis of secondary metabolites (55 DEPs, 33.33%, ko01110), phenylpropanoid biosynthesis (14 DEPs, 8.48%, ko00940), ribosome (14 DEPs, 8.48%, ko03010), carbon fixation in photosynthetic organisms (13 DEPs, 7.88%, ko00710) and glyoxylate and dicarboxylate metabolism (12 DEPs, 7.27%, ko00630) following behind, whereas cysteine and methionine metabolism (7 DEPs, 4.24%, ko00270), alanine, aspartate and glutamate metabolism (7 DEPs, 4.24%, ko00250) as well as flavonoid biosynthesis (5 DEPs, 3.03%, ko00941) demonstrated the fewest DEPs.

### 2.5. Screening of Candidate DEPs Associated with the Growth Inhibition of Lateral Branches

**Figure 6:**
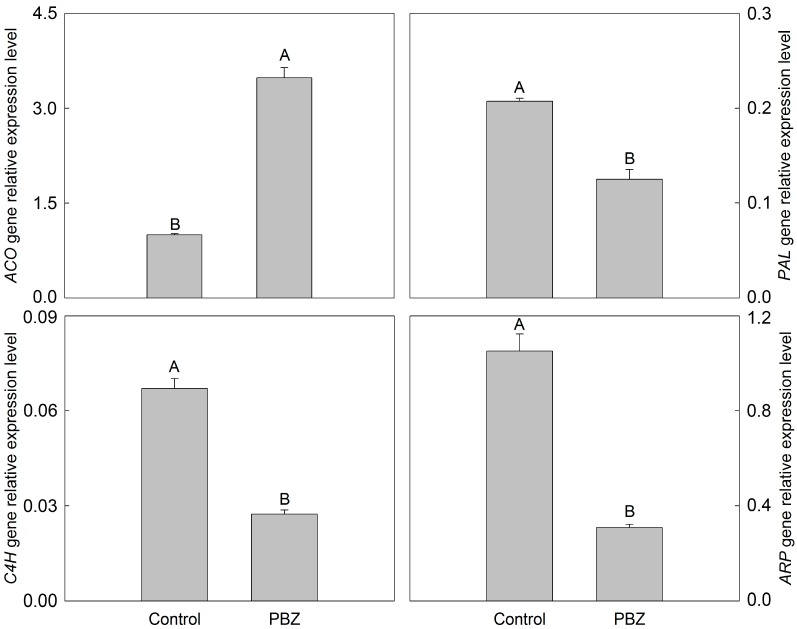
mRNA expression patterns of four representative DEPs in *P. lactiflora* lateral branches. Different letters indicate highly significant differences (*p* < 0.01).

According to the function and classification of the 178 identified DEPs, 34 candidate DEPs associated with the growth inhibition of *P. lactiflora* lateral branches were obtained, which could be classified into eight categories (Table S7). Among them, cell wall metabolism belonging to biosynthesis of secondary metabolites and phenylpropanoid biosynthesis demonstrated the largest number of DEPs (13 DEPs), followed by cell structure-related proteins (5 DEPs), defense and stress response (4 DEPs), hormone-related proteins (4 DEPs), energy metabolism (3 DEPs), carbohydrate transport and metabolism (2 DEPs) together with protein transport and metabolism (2 DEPs), while lipid transport and metabolism (1 DEPs) contained the fewest DEPs. As expected, the expression levels of DEPs in cell wall metabolism, cell structure-related proteins and energy metabolism all presented downtrends under PBZ application, whereas the opposite trends were observed in defense and stress response, carbohydrate transport and metabolism, lipid transport and metabolism together with protein transport and metabolism in comparison with the control. Of the 34 candidate DEPs, 1-aminocyclopropane-1-carboxylate oxidase (ACO), phenylalanine ammonia-lyase (PAL), and cinnamate 4-hydroxylase (C4H) were blasted to *P. lactiflora*, which had been submitted to NCBI by us, and auxin-repressed protein (ARP) was blasted to tree peony, which was the closest living relative of *P. lactiflora*. Therefore, the gene sequences corresponding to these 4 DEPs were amplified and their changes in the *P. lactiflora* lateral branches under control and PBZ application were verified. Gene expression patterns displayed that *PAL*, *C4H* and *ARP* were expressed lowly under PBZ application, whereas *ACO* presented the opposite trend (Figure 6), which was not consistent with the changes of the corresponding DEPs expression levels due to translational or post-translational regulation [19].

## 3. Discussion

iTRAQ technology is a powerful means of determining relative protein levels with high repeatability, and it is one of the most sensitive markers in the current studies of proteomics [20,21]. This technology has been widely used in life science research and made great achievements [15,18,19,22], but little information is available about the protein study of *P. lactiflora* using iTRAQ. In the present study, we found PBZ inhibited the growth of *P. lactiflora* lateral branches, including their length, diameter and the number of lateral buds. In order to further clarify the mechanism of PBZ inhibiting the lateral branch growth, iTRAQ technology was firstly used to compare the protein expression differences of *P. lactiflora* lateral branches under control and PBZ application. According to searching of the eudicotyledons database, 3733 proteins were obtained, and 178 DEPs were identified, including 98 up-regulated DEPs and 80 down-regulated DEPs. Thereafter, 34 candidate DEPs associated with the growth inhibition of *P. lactiflora* lateral branches were obtained according to their function and classification. To better understand the molecular mechanism of PBZ inhibiting the growth of lateral branches, these 34 candidate DEPs were classified into eight categories, their functions and regulation in inhibiting the growth of lateral branches were discussed in the below.

### 3.1. Defense and Stress Response

In this study, four identified DEPs were classified into this category, including lipoxygenase (LOX), peroxiredoxin-2E (PRX2E), catalase (CAT) and heat-shock protein (HSP). Under adversity stress, a plant can perceive the stress signal, and reactive oxygen species (ROS) are produced due to lipid peroxidation catalyzed by LOX, as well as a large amount of H_2_O_2_ is accumulated due to the enhanced ROS metabolism. Moreover, both ROS and H_2_O_2_ impair the cells and accelerate cell aging and disintegration [23]. ROS can be scavenged by PRX2E, and H_2_O_2_ can be reduced to H_2_O by CAT [24,25]. In *P. lactiflora* lateral branches, the expression levels of LOX, PRX2E and CAT were all increased under PBZ application, suggesting that *P. lactiflora* suffered from PBZ stress and both PRX2E and CAT were important protective enzymes in plants.

HSP is a type of stress protein in plants induced by high temperature, heavy metal ions and other adverse environmental factors. It is believed that HSP plays a key part in reducing the damage caused by adversity stress [26]. In this study, the expression level of HSP was also enhanced to protect lateral branches under PBZ application, which was consistent with the reports in *Primula* [27].

### 3.2. Hormone-Related Proteins

The growth and development of lateral branches involves a variety of changes in hormone levels. In this category, ACO, ARP, isopentenyl diphosphate isomerase (IPI) and geranylgeranyl pyrophosphate synthase (GGPS) were identified. ACO catalyzes the synthesis of ethylene, and *ACO* is widely accepted as the rate-limiting gene [28]. When *P. lactiflora* was treated with PBZ, the expression level of ACO was up-regulated, which could increase the content of ethylene inducing the senescence and abscission of lateral branches and finally inhibiting their growth.

The physiological role of PBZ was primarily to accelerate the decomposition of IAA and hinder the biosynthesis of gibberellin (GA), which slowed and inhibited the vegetative growth of plants [29]. Similar results were found in this study. ARP, an auxin-repressed protein, was up-regulated under PBZ application, which revealed that the content of IAA in *P. lactiflora* could be significantly reduced, which enabled the lateral buds to elongate normally, but stop their growth and maintain a dormant state. Similar results were reported in shoot elongation of black locus [30]. Moreover, the down-regulated expression levels of IPI and GGPS involved in the biosynthesis of GA could decrease GA synthesis, which also could not promote the growth of lateral branches.

### 3.3. Carbohydrate Transport and Metabolism

Aquaporins (AQPs) are a type of membrane proteins that mediate water transport through the membrane and play an important role in maintaining cell osmotic balance [31]. In this category, only two AQPs were identified, one belonged to the plasma membrane intrinsic proteins (PIPs) and the other could not be identified. Liu *et al.* [32] found that the *AQP1* from wintersweet was expressed most heavily in the leaves under high temperature stress. The study of Ahamed *et al.* [33] demonstrated that PIPs controlled rice root water uptake functions in a cold acclimation process. Similarly, the expression levels of our identified AQP and PIP1-2 were higher in *P. lactiflora* lateral branches under PBZ application than those of control, suggesting that *P. lactiflora* lateral branches controlled the expression of AQPs to restore normal growth, which provided water for resuming their growth.

### 3.4. Lipid Transport and Metabolism

Phospholipase D (PLD), one of the key enzymes of hydrolyzing phospholipids in cell membranes, plays a key part in maintaining the structure, function and stability of cell membranes [34]. Li *et al.* [35] found that UV-B irradiation induced the degradation of membrane lipids, and the degradation of membrane lipids in PLD δ-knockout plants was more severe than that in wild type plants. Under PBZ application, *P. lactiflora* also experienced stress; meantime, the up-regulated PLD in lateral branches could induce the hydrolysis of numerous phospholipids in cell membranes, which damaged the structure and function of the cell membrane, resulting in the inhibited growth of the lateral branches.

### 3.5. Protein Transport and Metabolism

Proteasome is responsible for the degradation of abnormal or damaged proteins [36]. In this category, two forms, a 20S proteasome subunit α type 7 (PSA7) as well as an ATP-dependent 26S proteasome regulatory subunit (PRS) were obtained, and they were expressed heavily under PBZ application, which was consistent with tea leaves under polyethylene glycol stress [37]. This meant that the increased proteasome activity maintained the growth of the lateral branched by degrading these proteins.

### 3.6. Energy Metabolism

Embden meyerhof generally existing in the organism can provide energy for the biological activity of an organism [29]. In this study, pyruvate kinase isozyme A (PKIA), phosphoglycerate kinase (PGK) and glyceraldehyde-3-phosphate dehydrogenase (GAPDH) in the embden meyerhof pathway were identified, which were the key enzymes in the process of triose oxidation. These proteins were lowly expressed in *P. lactiflora* lateral branches under PBZ application, which implied that *P. lactiflora* rarely provided energy for the growth of lateral branches.

### 3.7. Cell Structure-Related Proteins

The cell is the basic unit of organism structure and function. Under PBZ application, lateral branches were significantly affected including the average cell area and thickness of the sclerenchyma cell wall. Moreover, five cell structure-related proteins were identified, including xyloglucan endotransglucosylase (XT), isoflavone reductase (IFR), actin (ACTIN) and actin-3 (ACTIN3). XT catalyzes the transfer of xyloglucan, which has a key part in cell wall modification and the cell elongation process [38]. IFR is associated with the secondary cell wall thickening [39]. The expression levels of XT and IFR in *P. lactiflora* lateral branches under PBZ application were all decreased, which might be closely related to secondary cell wall thinning.

ACTIN plays many important roles in cell shape, cell movement, cell junction, cytoplasmic streaming and cell contraction [40,41]. Two ACTIN were obtained in this study, and they were all down-regulated under PBZ application. These results revealed that the cell shape and cell division were affected, which was consistent with cell shrinkage and a smaller average cell area in cross section (Figure 2).

### 3.8. Cell Wall Metabolism

Cell wall plays important roles in maintaining cell shape, controlling cell growth, material transportation and information transfer, defense and resistance. In addition, polysaccharides (cellulose, hemi-cellulose, pectin, *etc.*), protein, lignin and other materials are the most important chemical components in the cell wall [29]. In the present study, 13 proteins related to cell wall metabolism were identified. Among of them, bifunctional 3-dehydroquinate dehydratase/shikimate dehydrogenase (DHD/SHD), PAL, C4H, cinnamoyl CoA reductase (CCR), cinnamyl alcohol dehydrogenase (CAD) and cinnamyl alcohol dehydrogenase 2 (CAD2) controlled the synthesis of lignin [42], pectinesterase/pectinesterase inhibitor 35-like (PE/PEI35L) inhibited methyl esterification of cell wall pectin [43], and sucrose synthase 2 (SUS2) was an integral component of the cellulose synthesis machinery [44]. Moreover, UDP-glucose pyrophosphorylase (UGPase) catalyzed the reversible reaction of glucose-1-pyrophosphate and UTP to UDP-glucose and pyrophosphate, and UDPG as the glucose donor was involved in the anabolism of sugar, cellulose, hemi-cellulose and pectin [45]. Under PBZ application, these proteins were all down-regulated, which was consistent with the results of our physiological and biochemical measurements (Figure 3), revealing that PBZ application interfered with the synthesis and accumulation of the main cell wall components and significantly affected the function of the cell wall, eventually inhibited the growth of *P. lactiflora* lateral branches.

## 4. Experimental Section

### 4.1. Plant Materials

A *P. lactiflora* cultivar “Zifengyu” with lots of lateral branches was taken as a plant material, which was cultivated in the germplasm repository of Horticulture and Plant Protection College, Yangzhou University, Jiangsu Province, China (32°30′N, 119°25′E). When their buds were exposed to the ground in March, foliar-spraying PBZ with 100 mg/mL was performed once a week until the withering stage of the flowers, whereas the control was treated with deionized water. After counting the number of lateral buds that grew well and could blossom in the full-bloom stage, the length and diameter of the lateral branches under the control and PBZ application were measured. These lateral branches were partly used for microstructure observation which were fixed in 3% glutaraldehyde, and the others were used for physiological measurement and quantitative proteomics analysis, which were immediately frozen in liquid nitrogen and then stored at −80 °C until analysis. Three biological replications with each 100 plants were performed when *P. lactiflora* was treated with PBZ application and control. Furthermore, there were three biological replications in morphological indices, microstructures and cell wall materials. But there were 100 equally mixed samples for the protein identification.

### 4.2. Microstructures Observation

The fixed top lateral branches of *P. lactiflora* were firstly washed three times with 0.1 mol/L phosphate buffer, and then dehydrated using a gradient ethanol solution (30%, 50%, 70%, 85%, 95% and 100%, 15 min each), treated with the mixture of acetone:anhydrous alcohol (1:1, 2:1, 1:0, *v*/*v*) (15 min), the mixture of acetone:isoamyl acetate (1:1, 1:2, *v*/*v*) (10 min) and pure isoamyl acetate (30 min). After the critical point drying and spraying gold using ion sputtering equipment (EIKO IB-3, Ibaraki, Japan) for 5 min, the environmental scanning electron microscopy (Philips XL-30 ESEM, Amsterdam, The Netherlands) was used to observe the samples.

### 4.3. Cell Wall Materials Fractionation and Determination

The cell wall materials were fractioned according to the method of Rose *et al.* [46] with some modifications. Anthrone–H_2_SO_4_ colorimetry was used to determine the cellulose content [47], the lignin content was measured according to the report of Müsel *et al.* [48] and the measurement of pectin content referred to the reports of Blumenkrantz *et al.* [49] and Majumder *et al.* [50].

### 4.4. Protein Extraction and Quantification

Lateral branches of *P. lactiflora* were firstly ground into powder, and then their total proteins were extracted from the freeze-dried powder using 500 μL lysis buffer (40 mM Tris-HCl, 7 M Urea, 4% CHAPS, 2 M Thiourea, pH 8.5) including 10 mM DTT, 2 mM EDTA and 1 mM PMSF. Subsequently, the resuspended powder was treated with 200 W ultrasonic for 15 min and 4 °C together with 25,000× *g* centrifugation for 20 min. Moreover, the mixture of supernatant and 5× volume of chilled acetone including 10% (*v*/*v*) TCA was performed, and then they were treated with −20 °C incubation for 2 h. After 4 °C and 16,000× *g* centrifugation for 20 min, the precipitate was transferred to another 1.5 mL centrifuge tube washing with lysis buffer including 10 mM DTT, 2 mM EDTA and 1 mM PMSF. After treatment with 200 W ultrasonic for 15 min and 4 °C together with 25,000 × *g* centrifugation for 20 min, in order to reduce the disulfide bonds in proteins and block the cysteines, 10 mM DTT and 55 mM IAM were separately added to the supernatant. Subsequently, the solution was treated with dark incubation for 45 min, and then the mixture of supernatant and 4× volume of chilled acetone was performed to precipitate proteins at −20 °C for 2 h. After 4 °C and 25,000× *g* centrifugation for 20 min as well as 5 min air-drying, 200 μL 0.5 M TEAB was used to dissolve the precipitate which was treated with 200 W ultrasonic for 15 min. Finally, the supernatant was transferred to a new tube after 4 °C and 25,000× *g* centrifugation for 20 min again. The Bradford method was used to quantify total protein and its integrity was assessed by 10% SDS-PAGE. The proteins in the supernatant were sent to the Beijing Genomic Institute (Shenzhen, China) and stored at −80 °C.

### 4.5. iTRAQ Labeling and Strong Cation Exchange (SCX) Fractionation

A sample of 100 μg total protein was treated with 37 °C digestion for 4 h via trypsin (protein/trypsin = 20:1). The digestion using trypsin was repeated at 37 °C for 8 h. Subsequently, vacuum centrifugation was used to dry the peptides which was reconstituted in 0.5 M TEAB and iTRAQ labeled by the manufacturer’s protocol for 8-plex iTRAQ reagent (Applied Biosystems, Foster, CA, USA). Finally, vacuum centrifugation was used to dry the labeled peptide mixtures again.

Next, a LC-20AB high-performance liquid chromatograph (HPLC) system (Shimazu, Kyoto, Japan) with an Ultremex SCX column (4.6 mm × 250 mm) (Phenomenex Inc., Torrance, CA, USA) was used to fractionate the samples. Four milliliters of buffer A (10 mM KH_2_PO_4_ in 25% ACN, pH 2.6) were used to reconstitute the iTRAQ-labeled peptide mixtures, subsequently, SCX separation was performed at a flow rate of 1 mL/min using 5% elution buffer B (25 mM NaH_2_PO_4_, 1 M KCl in 25% ACN, pH 2.7) for 7 min, followed by a linear gradient of 5%–60% buffer B for 20 min, 60%–100% buffer B in 2 min, maintenance at 100% buffer B for 1 min, and finally backed to 5% buffer B for 10 min equilibration. The measurement of the absorbance at 214 nm was used to monitor the elution, and 20 fractions were obtained after screening, a Strata X C18 column (Phenomenex Inc.) was used to desalt them and then vacuum-dried.

### 4.6. LC-ESI-MS/MS Analysis Based on Triple TOF 5600

Each fraction was resuspended to approximately 0.5 μg/μL using buffer A (5% ACN, 0.1% FA) and centrifuged at 20,000× *g* for 10 min to remove the insoluble substances. A 5 μL volume of the supernatant of each fraction was separated by a LC-20AD nanoHPLC (Shimadzu, Japan), and the details were as follows: the samples were loaded onto a C18 trap column in 4 min at 8 μL/min, and then the samples were eluted onto a 10 cm analytical C18 column (inner diameter 75 μm) at 300 nL/min, separated and transferred to an MS system; the eluted system was run starting from 5% buffer B (95% ACN, 0.1% FA) for 5 min, followed by a 35 min linear gradient of 5%–35% buffer B, 35%–60% buffer B for 5 min, 60%–80% buffer B for 2 min, maintenance at 80% B for 2 min, and finally returned to 5% for 1 min and equilibration for 10 min.

A TripleTOF 5600 System (AB SCIEX, Concord, ON, Canada) with an ion spray voltage of 2.5 kV, a curtain gas of 30 psi, a nebulizer gas of 15 psi, and an interface heater temperature of 150 °C was used to acquire data. The MS was operated with an RP of greater than or equal to 30,000 FWHM for the TOF MS scans. For IDA, 250 ms was used to collect survey scans, and up to 30 product ion scans were acquired if exceeding a threshold of 120 counts/s and with a 2+ to 5+ charge-state. Total cycle time was 3.3 s, and Q2 transmission window was 100 Da for 100%. Four time bins with the 40 GHz multichannel TDC detector with a four-anode channel detection were summed for each scan at a pulser frequency value of 11 kHz. All precursor ions for collision-induced dissociation were happened at a sweeping collision energy setting of 35 ± 5 eV with the addition of iTRAQ adjust rolling collision energy. 1/2 of the peak width (15 s) was used for dynamic exclusion, and the precursor was refreshed off the exclusion list.

### 4.7. Database Search and Quantification

Proteome Discoverer 1.2 (PD 1.2, Thermo Fisher Scientific, San Jose, CA, USA) was used to convert raw data files (sample.wiff) into the mgf format files (sample. mgf), and then Mascot search engine (Matrix Science, London, UK; version 2.3.02) was used to searched against core eudicotyledons database (Available online: http://www.ncbi.nlm.nih.gov/protein/?term=txid91827 (Organism:exp)) including 910,008 sequences. A mass tolerance of 0.05 Da was accepted for intact peptide masses and 0.1 Da for fragmented ions to identify protein, allowing one missed cleavages in the trypsin digests. The considered potential variable modifications contained Gln- > pyro-Glu (N-term Q), Deamidated (NQ) and Oxidation (M), and the fixed modifications contained Carbamidomethyl (C), iTRAQ8plex (K) and iTRAQ8plex (N-term). +2 and +3 were set as the charge states of peptides. Specifically, an automatic decoy database search was conducted in Mascot by choosing the decoy checkbox where a random sequence of database was generated and tested for raw spectra together with the real database. In order to decrease the probability of false peptide identification, only the significance scores (≥20) at the 99% confidence interval peptides were considered as the identified protein. Moreover, each confident protein contained at least one unique peptide. In order to quantify protein, at least two independent peptides were included in a protein. The median ratio in Mascot was used to weigh and normalize the quantified protein ratios, and differentially expressed proteins were only defined as those with fold changes >1.2 and *p*-values < 0.05.

### 4.8. Bioinformatics and Annotations

Blast2GO program [51] was used to perform the functional annotations of proteins against the non-redundant protein database (NR; NCBI, Bethesda, MD, USA; Available online: http://www.ncbi.nlm.nih.gov), and these identified proteins were also classified and grouped by matching the COG database (NCBI, Bethesda, MD, USA; Available online: http://www.ncbi.nlm.nih.gov/COG/). Moreover, the GO enrichment and KEGG pathway enrichment analysis were conducted.

### 4.9. Quantitative RT-PCR (Q-PCR) for Identified Proteins

Gene expression pattern was detected by Q-PCR technology via a BIO-RAD CFX96™ Real-Time System (Bio-Rad, Hercules, CA, USA). CTAB extraction protocol was modified to extract total RNA [52], and then 1% agarose gel electrophoresis and a spectrophotometer (Eppendorf, Hamburg, Germany) were used to confirm the integrity of RNA. Next, 1 µg RNA from each sample was used as templates to synthesize the cDNA with PrimeScript^®^ RT reagent Kit With gDNA Eraser (TaKaRa, Kyoto, Japan). In this study, the internal control was *P. lactiflora* *Actin* (JN105299) [53], and Table S8 listed all gene-specific primers. SYBR^®^ Premix Ex Taq™ (TaKaRa) was used to conduct the Q-PCR, and the 2^−∆∆*C*t^ comparative threshold cycle (*C*_t_) method was calculated the levels of gene relative expression [54], meanwhile, the *PAL* expression level of control was used as the control. A Bio-Rad CFX Manager V1.6.541.1028 software (Bio-Rad, Hercules, CA, USA) was used to gather the *C*_t_ values of three triplicate reactions.

**Figure 7:**
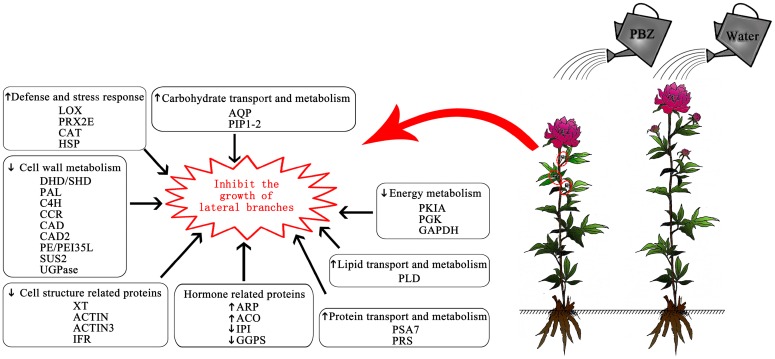
An overview of *P. lactiflora* lateral branches response to PBZ application. LOX: lipoxygenase; PRX2E: peroxiredoxin-2E; CAT: catalase; HSP: heat-shock protein; AQP: Aquaporin; PIP: plasma membrane intrinsic protein; DHD/SHD: dehydratase/shikimate dehydrogenase; PAL: phenylalanine ammonia-lyase; C4H: cinnamate 4-hydroxylase; CCR: cinnamoyl CoA reductase; CAD: cinnamyl alcohol dehydrogenase; PE/PEI35L: pectinesterase/pectinesterase inhibitor 35-like; SUS2: sucrose synthase 2; UGPase: UDP-glucose; PKIA: pyruvate kinase isozyme A; PGK: phosphoglycerate kinase; GAPDH: glyceraldehyde-3-phosphate dehydrogenase; PLD: Phospholipase D; XT: xyloglucan endotransglucosylase; ACTIN3: actin-3; IFR: isoflavone reductase; ARP: auxin-repressed protein; ACO: 1-aminocyclopropane-1-carboxylate oxidase; IPI: isopentenyl diphosphate isomerase; GGPS: geranylgeranyl pyrophosphate synthase; PSA7: proteasome subunit α type 7; PRS: proteasome regulatory subunit.

## 5. Conclusions

In conclusion, differential proteomic analysis of *P. lactiflora* lateral branches under PBZ application and control displayed significant changes in the biological processes. Figure 7 summarizes the major findings. When PBZ was applied in *P. lactiflora*, defense and stress response began to reduce the damage quickly, but the excessive accumulation of abnormal proteins made lateral branches degrade the damaged proteins by increasing the PSA7 and PRS expression levels in protein transport and metabolism, and water was transported to lateral branches by enhancing the AQP and PIP1-2 expression levels in carbohydrate transport and metabolism, which made the lateral branches restore normal growth. However, the increased PLD expression level in the lipid transport and metabolism and the decreased cell structure-related proteins (XT, ACTIN, ACTIN3 and IFR) expression levels caused the cell membrane and other cellular structures to be destroyed. Furthermore, a significant reduction of energy in the embden meyerhof pathway (decreased PKIA PGK and GAPDH expression levels) was produced, and the synthesis of the cell wall materials was blocked (decreased DHD/SHD, PAL, C4H, CCR, CAD, CAD2, PE/PEI35L, SUS2 and UGPase expression levels). In addition, the contents of IAA (increased ARP expression level) and GA (decreased IPI and GGPS expression levels) promoting the growth of lateral branches were reduced, and ethylene (increased ACO level) causing the lateral branches to age was produced in abundance, eventually inhibiting the growth of lateral branches. These results provide a theoretical basis for removing *P. lactiflora* lateral branches using PBZ.

## Supplementary Materials

Supplementary materials can be found at http://www.mdpi.com/1422-0067/16/10/24332/s1.
