# Supplementary material for: Quantitative Proteomics Analysis of Herbaceous Peony in Response to Paclobutrazol Inhibition of Lateral Branching
Source: Int J Mol Sci. 2015 Oct 14;16(10):24332–52. doi: 10.3390/ijms161024332 (PMC4632753; doi:10.3390/ijms161024332)
Supplement: Supplementary file 1 [file ijms-16-24332-s001.zip › ijms-91623-Supplementary Information.pdf]

Supplementary Information

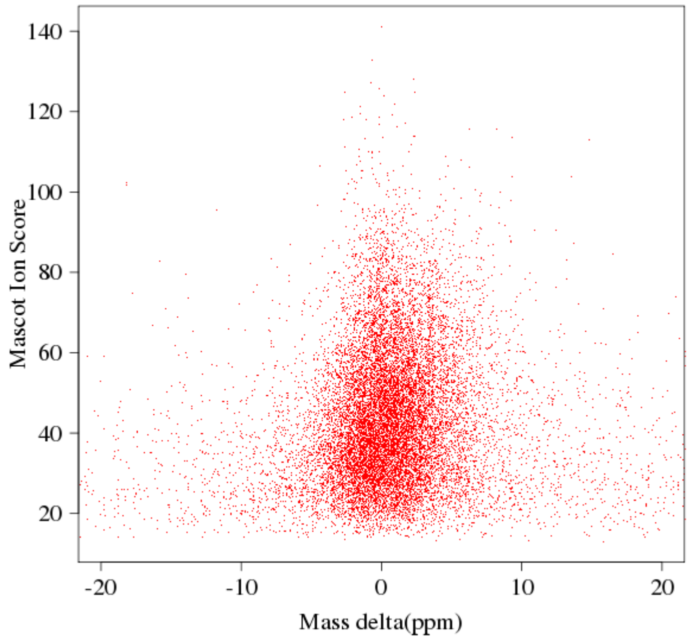

Figure S1. The error distribution of the peptide spectra match quality in *P. lactiflora* lateral branches.

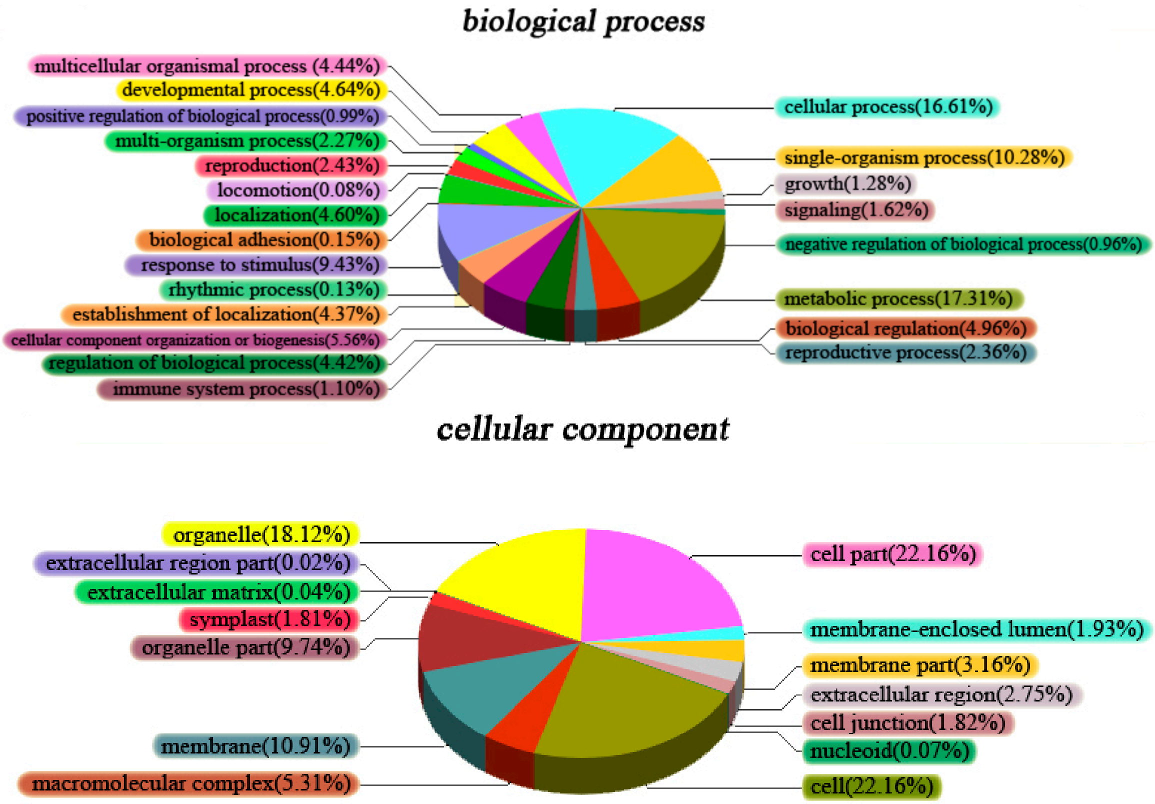

Figure S2. *Cont.*

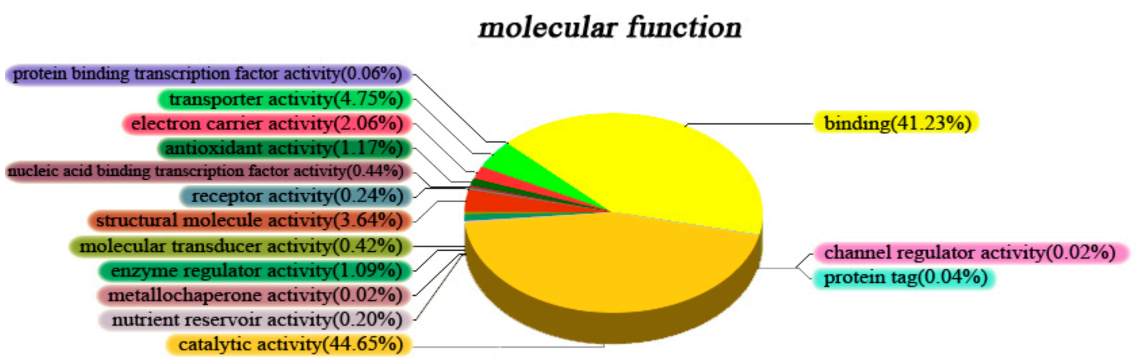

Figure S2. The GO analysis of total identified proteins in *P. lactiflora* lateral branches. GO: gene ontology.

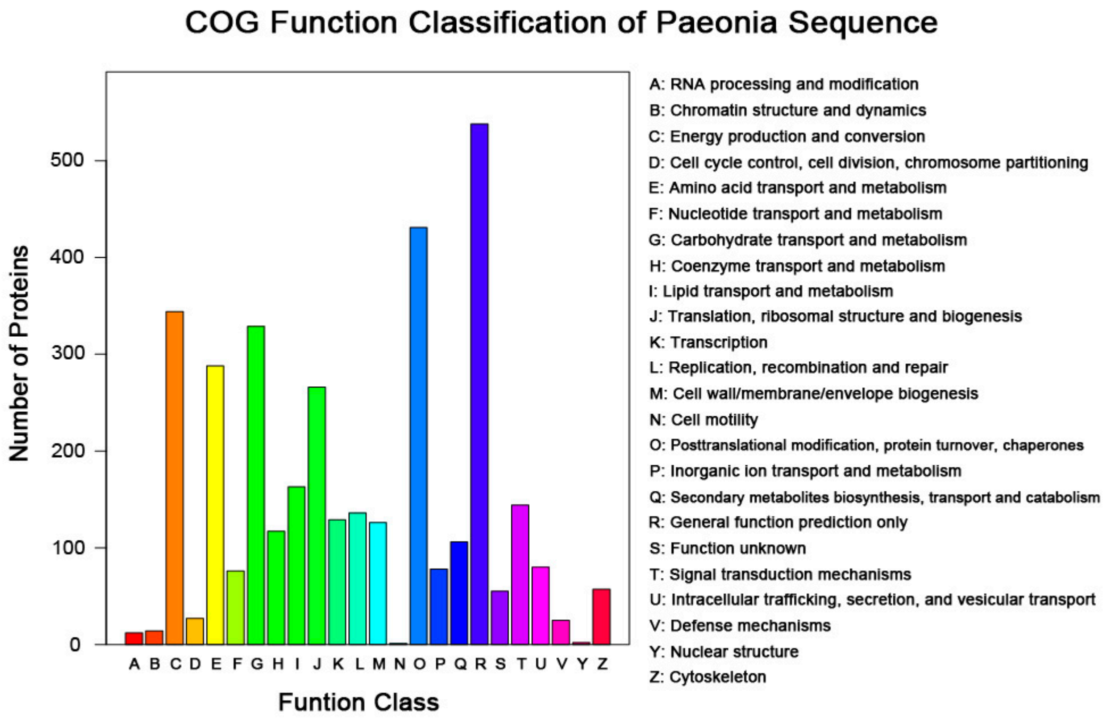

Figure S3. The COG analysis of total identified proteins in *P. lactiflora* lateral branches. COG: cluster of orthologous groups of proteins.

Table S6. 74 KEGG pathways with pathway ID information.

No.	Pathway	DEPs with Pathway Annotation (165)	Pathway ID
1	Metabolic pathways	90 (54.55%)	ko01100
2	Biosynthesis of secondary metabolites	55 (33.33%)	ko01110
3	Phenylpropanoid biosynthesis	14 (8.48%)	ko00940
4	Glyoxylate and dicarboxylate metabolism	12 (7.27%)	ko00630
5	Carbon fixation in photosynthetic organisms	13 (7.88%)	ko00710
6	Ribosome	14 (8.48%)	ko03010
7	Cysteine and methionine metabolism	7 (4.24%)	ko00270
8	Tryptophan metabolism	3 (1.82%)	ko00380
9	One carbon pool by folate	4 (2.42%)	ko00670

Table S6. *Cont.*

No.	Pathway	DEPs with Pathway Annotation (165)	Pathway ID
10	Flavonoid biosynthesis	5 (3.03%)	ko00941
11	Alanine, aspartate and glutamate metabolism	7 (4.24%)	ko00250
12	Photosynthesis—antenna proteins	3 (1.82%)	ko00196
13	Propanoate metabolism	5 (3.03%)	ko00640
14	Isoflavonoid biosynthesis	2 (1.21%)	ko00943
15	Pentose and glucuronate interconversions	5 (3.03%)	ko00040
16	Vitamin B6 metabolism	3 (1.82%)	ko00750
17	Glycolysis/Gluconeogenesis	10 (6.06%)	ko00010
18	Histidine metabolism	2 (1.21%)	ko00340
19	Arginine and proline metabolism	6 (3.64%)	ko00330
20	Phenylalanine metabolism	6 (3.64%)	ko00360
21	Nitrogen metabolism	4 (2.42%)	ko00910
22	Linoleic acid metabolism	2 (1.21%)	ko00591
23	Purine metabolism	7 (4.24%)	ko00230
24	Glycine, serine and threonine metabolism	6 (3.64%)	ko00260
25	Peroxisome	5 (3.03%)	ko04146
26	Amino sugar and nucleotide sugar metabolism	7 (4.24%)	ko00520
27	Ascorbate and aldarate metabolism	5 (3.03%)	ko00053
28	Cyanoamino acid metabolism	3 (1.82%)	ko00460
29	Lysine degradation	2 (1.21%)	ko00310
30	Galactose metabolism	4 (2.42%)	ko00052
31	Selenocompound metabolism	2 (1.21%)	ko00450
32	Stilbenoid, diarylheptanoid and gingerol biosynthesis	2 (1.21%)	ko00945
33	Glycerolipid metabolism	2 (1.21%)	ko00561
34	Phenylalanine, tyrosine and tryptophan biosynthesis	3 (1.82%)	ko00400
35	Flavone and flavonol biosynthesis	1 (0.61%)	ko00944
36	Pyruvate metabolism	5 (3.03%)	ko00620
37	Terpenoid backbone biosynthesis	2 (1.21%)	ko00900
38	Riboflavin metabolism	1 (0.61%)	ko00740
39	Tyrosine metabolism	3 (1.82%)	ko00350
40	beta-Alanine metabolism	2 (1.21%)	ko00410
41	Glycosphingolipid biosynthesis—ganglio series	1 (0.61%)	ko00604
42	Glycosaminoglycan degradation	1 (0.61%)	ko00531
43	Proteasome	3 (1.82%)	ko03050
44	Fatty acid metabolism	3 (1.82%)	ko00071
45	Ether lipid metabolism	1 (0.61%)	ko00565
46	Valine, leucine and isoleucine degradation	2 (1.21%)	ko00280
47	Sphingolipid metabolism	1 (0.61%)	ko00600
48	Limonene and pinene degradation	1 (0.61%)	ko00903
49	Lysine biosynthesis	1 (0.61%)	ko00300
50	Plant hormone signal transduction	3 (1.82%)	ko04075
51	RNA degradation	3 (1.82%)	ko03018
52	Photosynthesis	4 (2.42%)	ko00195
53	Isoquinoline alkaloid biosynthesis	1 (0.61%)	ko00950
54	Porphyrin and chlorophyll metabolism	1 (0.61%)	ko00860

Table S6. *Cont.*

No.	Pathway	DEPs with Pathway Annotation (165)	Pathway ID
55	alpha-Linolenic acid metabolism	2 (1.21%)	ko00592
56	Starch and sucrose metabolism	5 (3.03%)	ko00500
57	Phagosome	3 (1.82%)	ko04145
58	Butanoate metabolism	1 (0.61%)	ko00650
59	Tropane, piperidine and pyridine alkaloid biosynthesis	1 (0.61%)	ko00960
60	mRNA surveillance pathway	2 (1.21%)	ko03015
61	Glycerophospholipid metabolism	1 (0.61%)	ko00564
62	Citrate cycle (TCA cycle)	2 (1.21%)	ko00020
63	Aminoacyl-tRNA biosynthesis	2 (1.21%)	ko00970
64	Other glycan degradation	1 (0.61%)	ko00511
65	Ribosome biogenesis in eukaryotes	1 (0.61%)	ko03008
66	Glutathione metabolism	2 (1.21%)	ko00480
67	Fructose and mannose metabolism	2 (1.21%)	ko00051
68	Ubiquitin mediated proteolysis	1 (0.61%)	ko04120
69	Pyrimidine metabolism	1 (0.61%)	ko00240
70	Plant-pathogen interaction	2 (1.21%)	ko04626
71	Endocytosis	1 (0.61%)	ko04144
72	Protein processing in endoplasmic reticulum	2 (1.21%)	ko04141
73	RNA transport	1 (0.61%)	ko03013
74	Oxidative phosphorylation	1 (0.61%)	ko00190

Table S7. Candidate DEPs involved in the growth inhibition of *P. lactiflora* lateral branches under PBZ application.

Accession	Species	Description	Abbreviation	Coverage (%)	Mass (Da)	Ratio (PBZ/Control)
		**Defense and stress response**				
gi|223547351	*Ricinus communis*	lipoxygenase	LOX	1.2	121,118	1.641
gi|557104863	*Arabidopsis thaliana*	peroxiredoxin-2E	PRX2E	12.3	29,350	1.622
gi|67008247	*Andrographis paniculata*	catalase	CAT	22.8	36,540	1.256
gi|223540303	*Ricinus communis*	heat-shock protein	HSP	9.3	22,372	1.593
		**Hormone related proteins**				
gi|375314636	*Paeonia lactiflora*	1-aminocyclopropane-1-carboxylate oxidase	ACO	11.9	44,259	1.321
gi|158564566	*Paeonia suffruticosa*	auxin-repressed protein	ARP	19.8	16,150	1.262
gi|350538405	*Solanum lycopersicum*	isopentenyl diphosphate isomerase	IPI	14.5	32,568	0.392
gi|75250205	*Hevea brasiliensis*	geranylgeranyl pyrophosphate synthase	GGPS	3.8	48,208	0.666
		**Carbohydrate transport and metabolism**				
gi|17017255	*Nicotiana tabacum*	aquaporin	AQP	8.4	34,867	1.499
gi|150416573	*Gossypium hirsutum*	aquaporin PIP1-2	PIP1-2	8.4	35,692	1.638
		**Lipid transport and metabolism**				
gi|1698844	*Ricinus communis*	phospholipase D	PLD	12.5	105,167	1.339
		**Protein transport and metabolism**				
gi|343172102	*Silene latifolia*	20S proteasome subunit alpha type 7	PSA7	16.5	33,107	1.217
gi|222842473	*Populus trichocarpa*	ATP-dependent 26S proteasome regulatory subunit	PRS	27.9	53,879	1.269
		**Energy metabolism**				
gi|225432854	*Vitis vinifera*	pyruvate kinase isozyme A	PKIA	11.1	72,564	0.769
gi|356525744	*Glycine max*	phosphoglycerate kinase	PGK	18	53,925	0.721
gi|355485086	*Medicago truncatula*	glyceraldehyde-3-phosphate dehydrogenase	GAPDH	11.7	53,435	0.414
		**Cell structure related proteins**				
gi|82394883	*Gerbera jamesonii*	xyloglucan endotransglucosylase	XT	8.4	39,213	0.711
gi|3287956	*Glycine max*	actin-3	ACTIN3	31.9	48,198	0.517
gi|527182282	*Genlisea aurea*	actin	ACTIN	33.3	36,707	0.504
gi|359475114	*Vitis vinifera*	isoflavone reductase	IFR	5.2	33,592	0.687
gi|223540897	*Ricinus communis*	isoflavone reductase	IFR	3.8	42,856	0.503

Table S7. *Cont.*

Accession	Species	Description	Abbreviation	Coverage (%)	Mass (Da)	Ratio (PBZ/Control)
		**Cell wall metabolism**				
gi|470133240	*Fragaria vesca*	bifunctional 3-dehydroquinate dehydratase/ shikimate dehydrogenase	DHD/SHD	4.1	68,103	0.78
gi|67867090	*Ulmus americana*	phenylalanine ammonia-lyase	PAL	2.9	67,303	0.358
gi|385718959	*Paeonia lactiflora*	phenylalanine ammonia-lyase	PAL	22.7	89,039	0.341
gi|285028552	*Fagopyrum esculentum*	phenylalanine ammonia-lyase	PAL	11.2	90,728	0.321
gi|363541683	*Medicago sativa*	phenylalanine ammonia-lyase	PAL	8.7	63,676	0.308
gi|459353590	*Paeonia lactiflora*	cinnamate 4-hydroxylase	C4H	33.9	68,846	0.448
gi|31414896	*Fragaria x ananassa*	cinnamoyl CoA reductase	CCR	5.6	46,058	0.504
gi|227286085	*Codonopsis lanceolata*	cinnamoyl CoA reductase	CCR	8.3	45,020	0.425
gi|347730541	*Quercus suber*	cinnamyl alcohol dehydrogenase 2	CAD2	8.2	31,545	0.462
gi|62734979	*Linum usitatissimum*	cinnamyl alcohol dehydrogenase	CAD	5.5	35,894	0.405
gi|470121238	*Fragaria vesca*	pectinesterase/pectinesterase inhibitor 35-like	PE/PEI35L	2.4	71,889	0.448
gi|541135565	*Betula luminifera*	sucrose synthase 2	SUS2	9.2	106,948	0.378
gi|213868495	*Ipomoea batatas*	UDP-glucose pyrophosphorylase	UGPase	8.5	64,370	0.48

Table S8. Gene-specific primers sequence for detection by Q-PCR.

Gene	Forward Primer (5′–3′)	Reverse Primer (5′–3′)
Actin	GCAGTGTTCCCCAGTATT	TCTTTTCCATGTCATCCC
ACO	GCCTCATCTTACTCTTCCAA	GCAAGCGACATCCTATTACC
PAL	TGCCTCGCTACTTACCCT	GCTCCTCCTCAAATGCTG
C4H	GCACGACAGGAGTAACAA	CACTCAATGGACCACAGC
ARP	GAGGTCGCTTTCTATGCC	AGATTGCTGCCAGGGTTG
